# Study of patterns of inheritance of premature ovarian failure syndrome carrying maternal and paternal premutations

**DOI:** 10.1186/s12881-018-0634-5

**Published:** 2018-07-09

**Authors:** Artur Beke, Henriett Piko, Iren Haltrich, Veronika Karcagi, Janos Rigo, Maria Judit Molnar, György Fekete

**Affiliations:** 1 1st Department of Obstetrics and Gynecology, Semmelweis University, Baross u. 27, Budapest, 1428 Hungary; 2 Institute of Genomic Medicine and Rare Disorders, Semmelweis University, Budapest, Hungary; 3 2nd Department of Pediatrics, Semmelweis University fekete, Budapest, Hungary

**Keywords:** Premature ovarian failure, Fragile X syndrome., Fragile X-associated tremor/ataxia syndrome., Trinucleotide expansion syndrome.

## Abstract

**Background:**

Premature ovarian failure / primary ovarian insufficiency (POF/POI) associated with the mutations of the FMR1 (Fragile-X Mental Retardation 1) gene belongs to the group of the so-called trinucleotide expansion diseases. Our aim was to analyse the relationship between the paternally inherited premutation (PIP) and the maternally inherited premutation (MIP) by the examination of the family members of women with POF, carrying the premutation allele confirmed by molecular genetic testing.

**Methods:**

Molecular genetic testing was performed in the patients of the 1st Department of Obstetrics and Gynecology with suspected premature ovarian failure. First we performed the southern blot analyses and for the certified premutation cases we used the Repeat Primed PCR.

**Results:**

Due to POF/POI, a total of 125 patients underwent genetic testing. The FMR1 gene trinucleotide repeat number was examined in the DNA samples of the patients, and in 15 cases (12%) deviations (CGG repeat number corresponding to premutation or gray zone) were detected. In 6 cases out of the 15 cases the CGG repeat number fell within the range of the so-called gray zone (41–54 CGG repeat) (4.8%, 6/125), and the FMR1 premutation (55–200 CGG repeat) ratio was 7.2% (9/125). In 4 out of the 15 cases we found differences in both alleles, one was a premutation allele, and the other allele showed a repeat number belonging to the gray zone. Out of 15 cases, only maternal inheritance (MIP) was detected in 2 cases, in one case the premutation allele (91 CGG repeat number), while in the other case an allele belonging to the gray zone (41 CGG repeat number) were inherited from their mothers. In 10 out of 15 cases, the patient inherited the premutation allele only from the father (PIP). In 5 out of the 10 cases (50%) the premutation allele was inherited from the father, and the repeat number ranged from 55 to 133. Out of 125 cases, 9 patients had detectable cytogenetic abnormalities (7.2%).

**Conclusions:**

The RP-PCR method can be used to define the smaller premutations and the exact CGG number. Due to the quantitative nature of the RP-PCR, it is possible to detect the mosaicism as well.

## Background

The FMR1 gene mutation is an associated disease, belonging to the so-called trinucleotid expansion diseases group. These diseases, FRAXA (Fragile X syndrome), POF/POI (premature ovarian failure) and FXTAS (tremor/ataxia syndrome) are associated with a CGG repeats expansion, which is located at the FMR1 gene promoter region. There are two pathological FMR1 alleles: premutation alleles have 55–200 repeats and full mutation alleles have > 200 repeats [1].The premutation allele is associated with the premature ovarian failure and the tremor/ataxia syndrome. The full mutation is responsible for the formation of the X-linked dominant inherited fragile X syndrome (Martin-Bell syndrome) [1].

The FMR1 gene encodes the protein FMRP, which is an RNA binding protein and plays a significant role in the transport processes of RNA, stabilization of RNA molecules, and mRNA translation. The FMRP protein is expressed in all tissues, but it was detected especially in the brain, ovary and testis tissues in significant amounts. Studies proved in fruit flies and mice, that due to the lack of the FMRP protein some translational disturbances occur, which affect the early neuronal development, neurotransmission processes and synaptic connections.

The premutation and full mutation cases are not only different in respect of the phenotype, but also the expression of the FMRP protein. The hyper-expression of the FMRP protein was confirmed in cases of premutation allele carriers, and the presence of the increased level of the CGG repeat transcripts have a significant role in the development of the phenotype.

In cases of the full mutation carrier FRAXA patients, the high CGG repeat numbers are in association with the heterochromatin condition, which results in the reduction/absence of the FMRP protein transcription. Experiments have shown that in case of the premutation allele, the transcription is characterised by active histone modification, while the full mutation allele is characterised by the hypermethylated condition, associated with hypoacetylated histone proteins.

Mutations of the FMR1 gene play a role in the development of three different conditions; the fragile X syndrome, premature ovarian failure and the tremor/ataxia syndrome (FXTAS). Patients carrying the full mutation allele are characterised by the fragile X syndrome. Children with fragile X syndrome have a range of cognitive impairments with boys typically more severely affected than girls. The premutation involvement of the FMR1 gene was described in 3–15% of the premature ovarian failure (POF), more recently known as primary ovarian insufficiency (POI) cases. POF/POI disease is a condition characterised by amenorrhoea, hypoestrogenism, elevated gonadotropin levels and infertility occurring in women before the age of 40 [2, 3].

Wheeler et al*.* also reported significantly higher rates of hot flashes or flushes in premutation carriers (15.4%) than controls (6.9%). CGG repeat size has been found to be associated with the risk of earlier menopause but in a non-linear manner, with the highest risk for those with mid-range repeats (approximately 70 to 100) [4].

The risk of POF increases if the CGG repeat number of the FMR1 gene exceeds 40, or if it falls in the so-called gray zone (41–54) [1]. More recent population studies have used 41–54 repeats. This discrepancy in the definition of the gray zone has become more important recently because several studies have now reported phenotypes associated with the gray zone or intermediate allele sizes. In 2006, it was recognized that premature ovarian insufficiency, which is associated with premutation expansions, is also present in gray zone expansion carriers [4]. Population screening studies also suggested that gray zone alleles are more highly represented in ataxia phenotypes in general, including multiple system atrophy, with rates as high as 8%. However, the prevalence rates are difficult to compare across studies given the different study designs, with varying types of ataxia patients included, mixed populations, differing ages, and lack of collected controls [5]. The involvement of certain gene defects in the POF disease shows wide variations, with the involvement of the FMR1 gene being described most frequently [1].

In carriers of the premutation allele the development of the tremor/ataxia syndrome (FXTAS) was described. The classic phenotype of the FXTAS is the kinetic tremor and cerebellar ataxia, and other symptoms include cognitive impairment, psychiatric disorders, peripheral neuropathy and autonomic nervous system problems. The symptoms start at about 50 years of age. The disease’s progression can show a great variety with a life expectancy around 5 to 25 years following the appearance of the symptoms [6]. 40–45% of the male premutation carriers and 8–16% of the female carriers showed tremor/ataxia syndrome, wherein the first symptoms were described at over 50 years of age. The background of the pathogenicity of the other phenotype associated with the premutation status, the tremor/ataxia syndrome (FXTAS), is still not fully understood [7–9].

The possible links between the premutation status of the POF/POI and tremor/ataxia syndrome FXTAS the FMR1 gene were explained with two theories.

One theory is that the protein FMRP, which is an RNA binding protein, encoded by the FMR1 gene, may be expressed in larger quantities in the premutation carriers, and have a suppressor effect on the translation of mRNAs encoded by a variety of other genes. Based on these, it is also believed, that the reduced transcription of the genes involved in the oocyte maturation might result in a reduced follicle stock [10, 11].

According to the other theory, the accumulating mutant FMR1 mRNA may have a long lasting toxic effect on the ovary and this results in the premature destruction of the follicles.

The expression of the toxic RNA can be associated with various clinical diseases, caused by both the proteins bound to the RNA, and the influence of the alternative splicing mechanism of other genes.

The increased FMRP protein levels result in neuronal toxicity, which can be detected in neurons and astrocytes as eosinophilic and ubiquitin positive nuclear inclusions in the cerebrum, thalamus, basal ganglia, cranial nerves and spinal cord [12, 13]. In the future the additional neurological examinations of the relatives carrying the premutation status can give an opportunity to detect any possible tremor/ataxia syndrome involvement. In addition, the neurological examinations of the premutation carrier female relatives could give an opportunity for the introduction of new test methods by mapping the differences with a potential diagnostic significance [14, 15]. We assess the neurological status of the parents by neurological examination and by using a special tremor testing device. Our results will be reported later in another publication.

The loss of AGG interruptions is thought to increase the probability of transmission of a full mutation allele. Yrigollen et al. studied the AGG interruption and *FMR1* CGG repeat allele stability during transmission and concluded that the risk of transmission by total length and AGG interruption show that the odds of transmission to a full mutation increase significantly with total length *(P* < 0.001). A subject with 0 AGG interruptions and a total length of 75 repeats would have an odds ratio 4.7 to 1 of having a full mutation expansion and a subject with 0 AGG interruptions and 76 repeats would have an odds ratio of 5.9 to 1 [16].

In European studies, in cases of POF/POI, the FMR1 premutation rate in the UK was 1.97% (5/254) and 10% in Italy (19/190) [17, 18]. In South America, the FMR1 gene premutation rate in an Argentine population was 3.76% for POF/POI (5/133) [19]. From Asian countries, the FMR1 premutation rates among POF/POI patients were 0.53% (2/379) in China [20], 1.56% in Japan (2/128) [21], and 2.14% (3/140) in India [22].

In the case of POF/POI, several researchers took into consideration the intermediate cases classified in the gray zone, in addition to the FMR1 premutation. Dean et al. found the gray zone incidence rate 3.57% (5/140) in an Indian population besides the FMR1 premutation (2.14%, 3/140) in POI cases [22]. Bodega et al. demonstrated 4.74% rate of gray zone cases (9/190) in Italian POF/POI patients with 10% FMR1 premutation rate (19/190) [18]. Guo et al. in China, with the occurrence of a lower FMR1 premutation (0.53%, 2/379), demonstrated 2.9% (11/379) incidence rate of the gray zone cases [20].

The aim of the study was to explore the frequency of expanded FMR1 alleles in women diagnosed with POF and in their parents to determine parental origin. Our aim was to analyse the relationship between the paternally inherited premutation (PIP) and the maternally inherited premutation (MIP) by the examination of the family members of women with POF, carrying the premutation allele, which was confirmed by molecular genetic testing.

Our study is the first genetic study in Hungary performed on premature ovarian failure / primary ovarian insufficiency (POF/POI) without previous population based study.

## Methods

The investigation process is the following: the patient must be involved in genetic counselling and clinical data should be included. If the patient suffers from POF disease, two blood samples are taken: one for the determination of hormone levels, and the other for molecular genetic and cytogenetic analyses. Applying the G-banding method we can determine the POF associated chromosome abnormalities. Our results on chromosome abnormalities will be reported in a publication later on.

The Southern blot analysis is suitable for determining the FMR1 gene premutation and full mutation status, and the RP-PCR can be used for the exact CGG repeat number analyses. At the confirmed premutation cases we also analyse the DNA sample of the parents if it is possible.

Patients of the 1st Department of Obstetrics and Gynaecology, Semmelweis University, with suspected premature ovarian failure (POF) were included in the study, after prior information and consent. The enrolment criteria were the following: secondary amenorrhoea, the ovaries stopped functioning before 40 years of age, FSH ≥ 40 IU/l in two different measurements and low estrogen levels. Exclusion criteria were if a patient had a surgery before, significantly affecting the follicle stock in both ovaries, or medication was used, damaging the ovarian function (chemotherapy).

Molecular genetic and cytogenetic (G-banding cytogenetic analyses) examinations were performed in cases of women with suspected premature ovarian failure. Southern blot analysis was performed during the molecular genetic analysis and the exact CGG repeat number was determined by the Repeat Primed polymerase chain reaction (RP-PCR) method.

DNA was isolated from blood samples of the patients, and the genomic DNA was digested with restriction endonucleases EcoRI and EagI at 37 °C all night long. Southern blot analysis was performed on the samples following the digestion, the samples were run on 0.5% agarose gel for 18 h at 40 V voltage. As a result, the DNA samples digested by restriction endonucleases, were separated by size. After the separation, the sample DNA was blotted from the agarose gel to the nitrocellulose membrane by the capillary principle. The membrane was probed with the radiolabeled Stb12.3 DNA probe (FMR1 gene specific), using hybridisation at 65 °C all night long. The principle of the method is random priming technique [23, 24]. The unbound radioactive probe was removed by incremental solutions (at 65 °C). Using a Packard Instant Imager device we performed the rapid measurement of the radioactive signals of the fragments, their preliminary assessment; then we put a Kodak XOMAT X-ray film on the membrane, and exposed at − 100 °C for 4–5 days. At this rating the normal fragment size in the healthy (normal) control group can be measured at 2.8 kb, and about at 5.2 kb in accordance with the inactive X. In the premutation cases the fragment size was measured between 2.9–3.3 and 5.3–5.7 kb.

The exact CGG repeat number was determined by the Repeat Primed (RP)-PCR technique. The genomic DNA sample was diluted to 20 ng/μl concentration, then we used 2 μl for the LCD reaction. The PCR reaction was performed with three primers, the FMR1 gene specific primers (forward and reverse, FAM-labeled) and a so-called CGG repeat primer together. The resulting PCR products were separated by capillary electrophoresis according to size, and the amount of the product can be determined based on the fluorescent signal intensity. The method is also good for identifying the number of AGG interruptions between CGG repeats.

## Results

Due to premature ovarian failure, a total of 125 patients underwent genetic testing. We examined the FMR1 gene trinucleotide repeat number in DNA samples of the patients, and in 15 cases (12%) we detected deviations (CGG repeat number corresponding to premutation or gray zone). In 6 cases out of the 15 cases the CGG repeat number fell within the range of the so-called gray zone (41–54 CGG repeat) (4.8%, 6/125), and the FMR1 premutation (55–200 CGG repeat) ratio was 7.2% (9/125).

In 4 cases out of 15 cases we found differences in both alleles, one was a premutation allele, and the other allele showed a repeat number belonging to the gray zone. (Table 1).

**Table 1:** In 6 cases the CGG repeat number was within the range 41–54 CGG repeat (gray zone), and in 9 cases we found FMR1 premutation (55–200 CGG repeat). In 4 cases we found differences in both alleles, one was a premutation allele, and the other allele showed a repeat number belonging to the gray zone

Family N^o^	Code	CGG repeat number with praemutation caused POF-POI	Allel number with premutation or gray zone	Premutation (PM) or gray zone (GZ)	Premutation with paternal (p) or maternal (m) origin	Original paternal allel	Original maternal allel	Repeat number expansion	AGG interruptions (n)	Full mutation in family	Family member with full mutation
1	191	30/91	1	PM	m	30	91	no	2	91-- > 200	son
2	252	41/55	2	PM and GZ	p (PM) m (GZ)	55	41	no	0		
3	258	22/79	1	PM	p	75	22	75-- > 79	1	75-- > 200	brother
4	276	22/42/52 (mosaic)	1	GZ	p	40	21	40-- > 42/ 40-- > 52	3		
5	296	50/82	2	PM and GZ	p (PM) m (GZ)	82	49	no	0		
6	311	52/76	2	PM and GZ	p (PM) m (GZ)	52/64/76 mosaic	52	no	3		
7	310	29/58	1	PM	p	58	29	no	3		
8	327	29/42	1	GZ	p	42	29	no	3		
9	365	22/46	1	GZ	p	46	23	no	3		
10	367	29/90	1	PM	p	88	30	88-- > 90	2		
11	377	23/45	1	GZ	p	45	23	no	3		
12	372	30/42	1	GZ	p	42	29	no	2		
13	375	23/41	1	GZ	m	23	41	no	3		
14	431	21/74	1	PM	p	70	21	70-- > 74	0		
15	436	30/89	1	PM	p	87	exit	87-- > 89	2	87-- > 200, 133-- > 200	other family member

FMR1 gene CGG repeat numbers associated with POF/POI, allels with premutations and grey zones, and paternal/maternal origin

Out of 125 cases 9 patients had detectable cytogenetic abnormalities (7.2%). No cases were found to have both chromosome abnormalities and expanded FMR1 alleles. About the findings of chromosome abnormalities we will report in another publication.

At the confirmed premutation and gray zone cases we completed the determination of the CGG repeat number of the FMR1 gene of the family members, and studied the maternal-paternal inheritance process.

Out of 15 cases, only maternal inheritance (MIP) was detected in only 2 cases, in one case the premutation allele (91 CGG repeat number), while in the other case an allele belonging to the gray zone (41 CGG repeat number) were inherited from the mother.

During the inheritance there was no expansion (CGG repeat number increase) observed (Table 1).

In 10 cases out of 15 cases, the patient inherited the premutation allele only from the father (PIP). In 5 out of 10 cases (50%), the premutation allele was inherited from the father, and the repeat number ranged from 55 to 133. Expansion of the repeat number was observed in 4 of these cases during the inheritance process (75 → 79, 88 → 90, 70 → 74, 87 → 89 CGG repeat number expansion). In three cases among the tested male relatives, the neurodegenerative differences in the presence of premutation or gray zone alleles were confirmed.

In 5 out of 10 cases (i.e. 50% of cases), the patient inherited a paternal allele with a repeat number within the gray zone. The repeat number varied between 41 and 46. Interestingly, in one case, the normal paternal allele showed mosaic expansion (40 repeat number), the patient had repeat numbers 22/42/52 in a mosaic form, meaning that most likely a 40 → 42 and a 40 → 52 repeat number expansion occurred during the inheritance of the paternal allele.

**Fig. 1:**
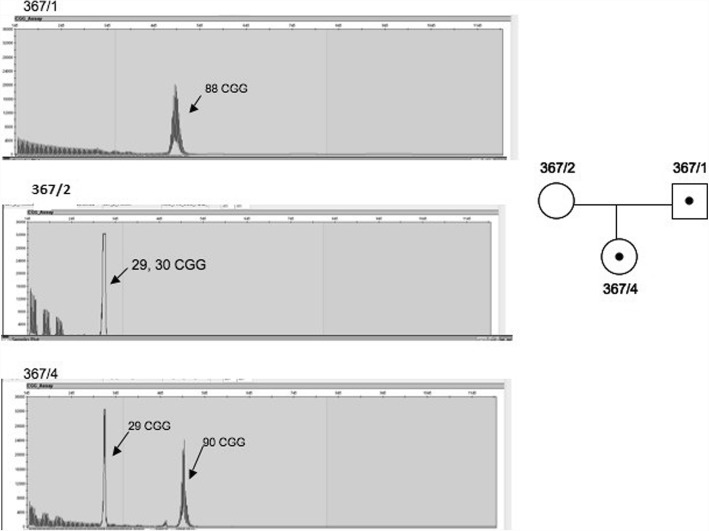
Paternal premutation allele inheritance (PIP) with low expansion (CGG repeat expansion 88 90). The figure shows the examination of the family No.10 (code 367). The patient inherited the normal 29 CGG repeat number allele from the mother. The other allele was inherited from the father, where the paternal 88 CGG repeat number expanded to 90 was observed in the patient. There was no evidence of AGG interruptions in the father. The two AGG interruptions are in accordance with the maternal allele

Figure 1. shows an example of the inheritance of the paternal premutation allele. We confirmed 88 CGG premutation repeat in the paternal sample (367/1) (code of the family/ code of the member of family), while the mother (367/2) carried the normal 29, 30 CGG repeats. Their daughter (367/4) inherited the normal 29 CGG maternal and the premutation CGG 90 paternal alleles. In this case the premutation paternal allele increased in the offspring by 2 CGG repeats (88 → 90 CGG repeat number expansion). (Fig. 1.)

**Fig. 2:**
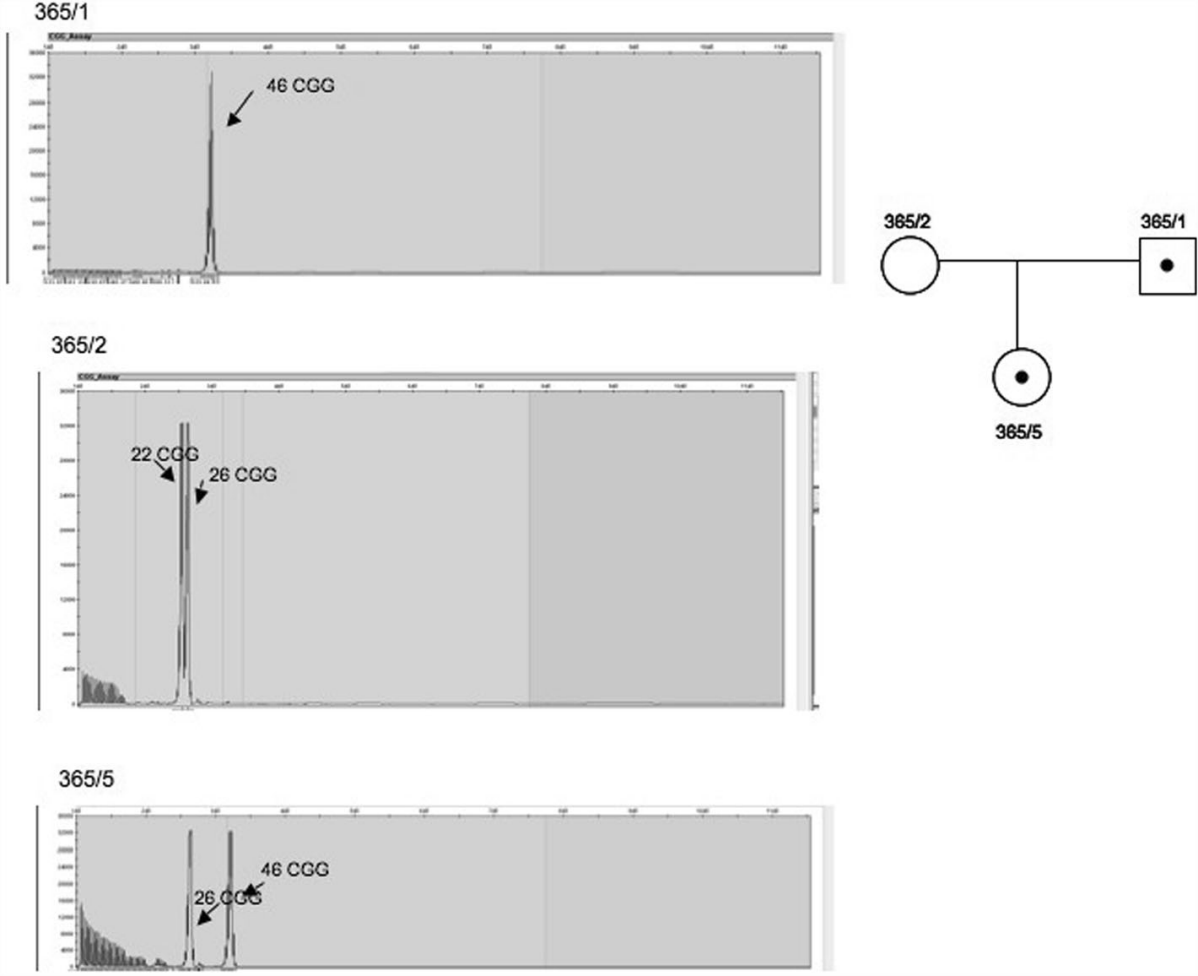
Inheritance of the paternal allele within the gray zone. The figure shows family No. 9 (code 365), the father (365/1) carries a 46 CGG repeat within the gray zone, the mother (365/2) carries the normal 22 and 26 CGG, and their daughter (365/5) carries the 26 and 46 CGG repeats

Figure 2. shows an example of the inheritance of the paternal allele within the gray zone.

In case of family No. 365 (Fig. 2), the father (365/1) carried the 46 CGG repeat within the gray zone, the mother (365/2) had the normal CGG 22 and 26 repeats, and their daughter (365/5) carried the 26 and 46 CGG repeats.

In 3 cases out of 15 cases maternal and paternal inheritance was simultaneously observed (PIP and MIP). In each case, the inherited paternal allele was a premutation allele, while the patient inherited the maternal allele with repeat numbers within the gray zone.

**Fig. 3:**
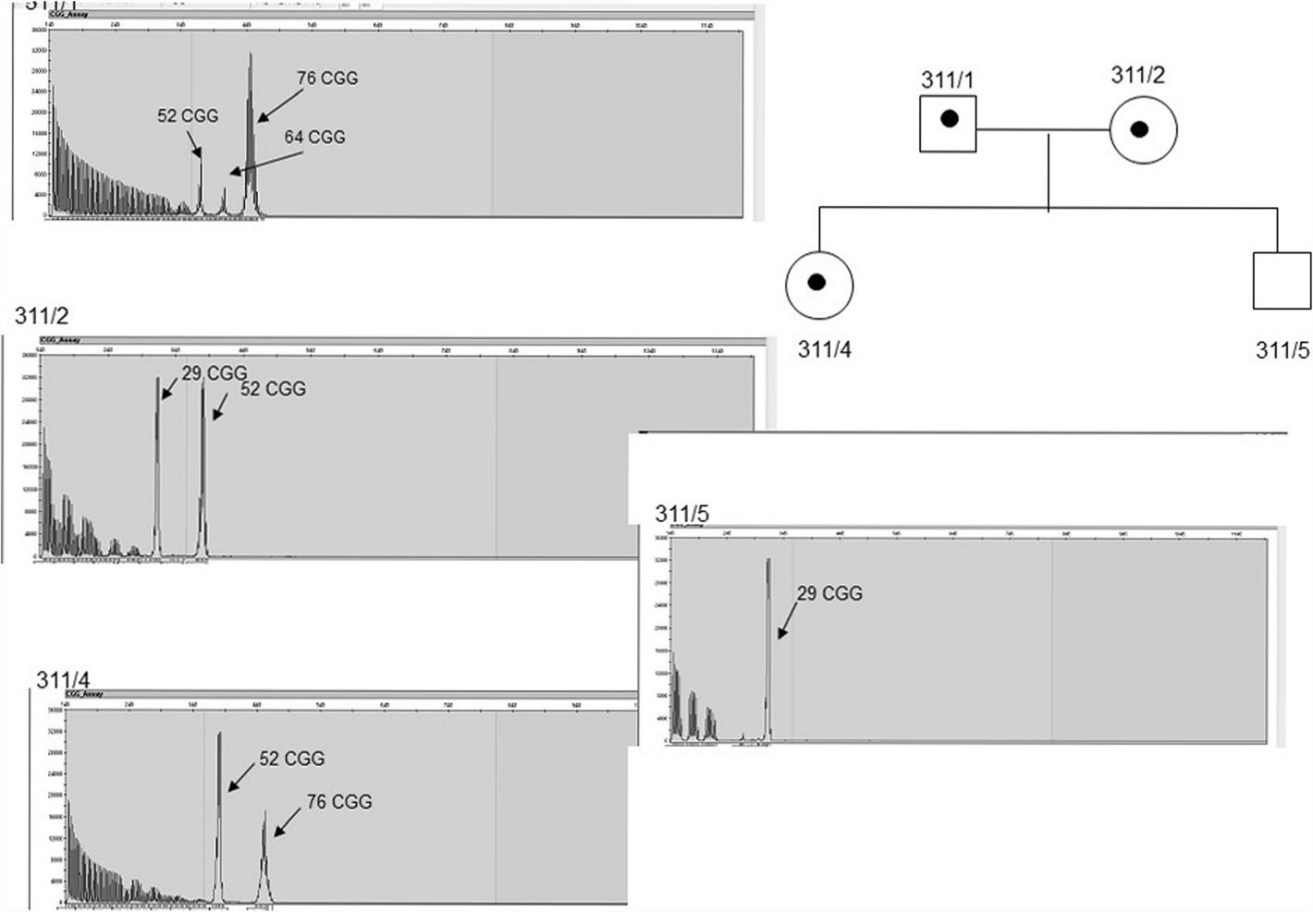
Paternal and maternal inheritance (PIP and MIP) with paternal mozaicism. The figure shows the family No.6 (code 311), where the maternal and paternal inheritance was observed in parallel (PIP and MIP). The patient inherited the paternal premutation allele, and the maternal allele with the repeat number within the gray zone. We detected mosaic form for the father with 52/64/76 CGG repeat numbers. The patient inherited the paternal 76 CGG repeat number allele, and the maternal 52 CGG repeat number allele

Interestingly, in one case we detected a mosaic form in the father, with the 52/64/76 CGG repeat numbers using RP-PCR method. The patient inherited the paternal allele with the 76 CGG repeat number, while the inherited maternal allele was the 52 CGG repeat number allele (Fig. 3).

In case of the mother (311/2) the 29 and 52 CGG repeats were detected, the 52 CGG fell within the gray zone. In the case of their daughter (311/4) we confirmed the maternal allele with the 52 and the paternal allele with the 76 CGG repeats, and thus we confirmed the premutation status as well. Her brother inherited the normal maternal allele carrying the 29 CGG repeat (Fig. 3).

In 3 families, the expansion rate of the CGG repeat number observed in the family exceeded 200, so full mutation occurred. In one case, the patient’s son, in another case his brother and in the third case other family members were affected by fragile X syndrome (Table 1).

**Fig. 4:**
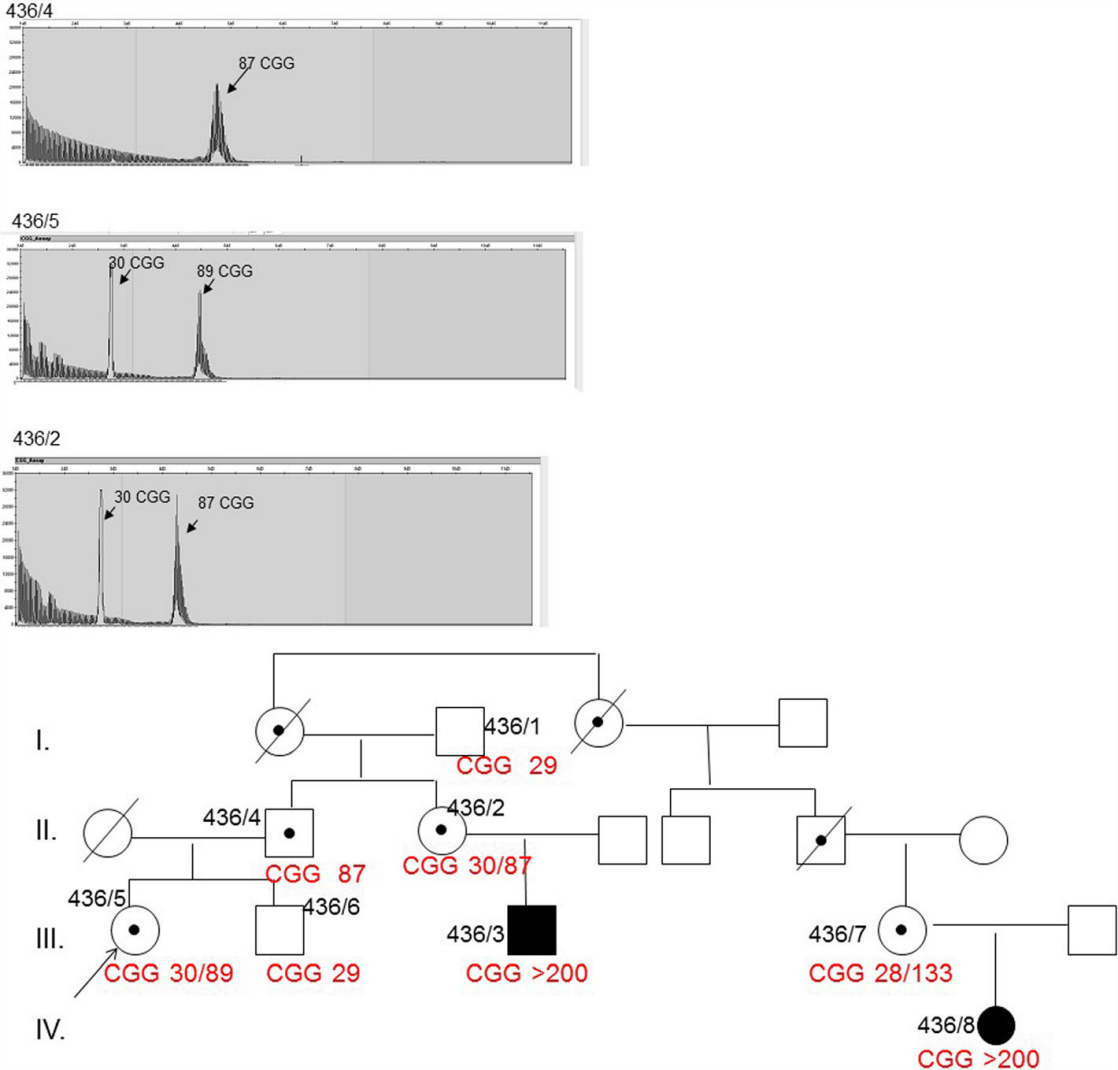
Full mutation occurrence in the family. Figure 4. shows the case of family No.15 (code 436), showing the Southern blot image in addition to the RP-PCR results. The patient (436/5) inherited the paternal 87 CGG premutation allele, the 89 CGG repeat showed a low repeat number expansion. There were two cases of full mutation form with the Fragile X syndrome in the family

Figure 4. shows the RP-PCR results and the image of the Southern blot. The father (436/4) carried the premutation 87 CGG repeat, the patient (436/5) inherited the paternal 87 CGG premutation allele, the 89 CGG repeat number showed a low repeat number expansion. The inherited maternal allele was a normal 29 CGG repeat allele. We analysed the sample of the sister of the father, and normal 30 CGG and premutation 87 CGG alleles were confirmed. There were two cases of full mutation form with the Fragile X syndrome in this family (Fig. 4.).

We investigated the presence of AGG interruptions playing a role in the repeat number expansion. The number of AGG interruptions was 3 in almost 50% of the cases, CGG repeat number expansion occurred in only one of these cases. If the number of AGG interruption was 0–2 (total of 8 cases), there were 4 cases of CGG repeat number expansion of the examined patients, and if the patients and their family members were taken into consideration together, the overall incidence of CGG repeat expansion was 7.

## Discussion

The primary aim of our research group was the clinical and molecular genetic analysis of women involved in the POF/POI disease, and the determination of the exact CGG repeat numbers with the new RP-PCR method. In the confirmed premutation cases we mapped the maternal and paternal inheritance process by examining the relatives.

In our material we detected 15 cases (12%) out of 125 cases, FMR1 premutation (9 cases) or gray zone (6 cases). We performed an FMR1 RP-PCR study in POF/POI patients for the first time in Hungary. If we examine the FMR1 premutation ratio, the observed 7.2% (9/125) in Europe is closer to the Italian results [18]. The ratio of the gray zone in POF/POI patients in our own material was 4.8% (6/125), which corresponded to the Italian results [18]. The ratio of the FMR1 premutation and gray zone detected by us was higher than in the Asian countries [20–22].

Based on our results, in the case of paternal inheritance, the FMR1 gene premutation CGG repeat number is inherited to the next generation with about the same number of repeats. In our studies we did not detect any CGG repeats rising to full mutation status in the offspring generation in paternal inheritance cases. In the examined samples, we detected premutation status in both the male and the female offspring.

In the case of maternal inheritance, the full mutation status is much more likely to occur in the offspring, depending on the premutation CGG number; and the number of the so-called AGG interruptions. The higher the premutation repeat number of repeats and the lack of AGG interruptions are, the more likely is that the full mutation status occurs in the next generation. The difference between the paternal and maternal inheritance is due to the difference between the male and female gamete maturation.

The determination of the number of AGG interruptions within the CGG repeat has a key role. If the number of AGG interruptions is 1 or 0, we have to consider the increase of the CGG repeat number occurring in the offspring, which definitely increases the risk of the FRAXA premutation or full mutation in the case of a mother carrying a gray zone (41–54 CGG repeat number). So we can say that during family planning the clinical geneticist should always consider the exact CGG repeat number, and also the number of AGG interruptions to give reliable results for a personalized risk assessment. Using the RP-PCR technique, these interruptions can be detected and quantified, for which we could predict the risk of CGG expansion possible in further generations. Therefore, in the premutation cases with higher CGG repeat numbers, the prenatal CGG repeat number diagnosis is definitely justified in order to determine the possible symptomatic fragile X syndrome.

Based on our experience, among the molecular genetic test methods the Southern blot analysis is suitable to detect the full mutation cases, higher premutation status, and to isolate the methylated and unmethylated X chromosome in the female specimens. The RP-PCR method can be used to define the smaller premutations and the exact CGG number. Due to the quantitative nature of the RP-PCR, it is possible to detect the mosaicism as well.

We found 15 cases of FMR1 gene premutations or gray zone (12%), beside 9 chromosome abnormalities (7.6%). Our findings are important and have significance for the clinical care of these patients during genetic counselling.

The limitation of the study is the few case numbers which can’t give a reliable genotype-phenotype correlation. Our study was the first genetic study in Hungary performed on premature ovarian failure / primary ovarian insufficiency (POF/POI) without other general population based study.

## Conclusions

The genetic examination of the premature ovarian failure is very important for the patient and her family also, because the genetic results have serious influence on the reproductive possibilities and family planning of the premutation carriers. The RP-PCR method can be used to define the smaller premutations and the exact CGG number. Due to the quantitative nature of the RP-PCR, it is possible to detect the mosaicism as well.

## Abbreviations

FMR1 gene: Fragile X Mental Retardation 1
FMRP: Fragile X Mental Retardation Protein
FRAXA: Fragile X Syndrome
FXTAS: Fraxa X Associated Tremor/Ataxia Syndrome
POF: Premature Ovarian Failure
POI: Primary Ovarian Insufficiency
RP-PCR: Repeat-Primed Polimerase Chain Reaction
